# Supplementary material for: EnzML: multi-label prediction of enzyme classes using InterPro signatures
Source: BMC Bioinformatics. 2012 Apr 25;13:61. doi: 10.1186/1471-2105-13-61 (PMC3483700; doi:10.1186/1471-2105-13-61)
Supplement: Addtional file 5 — The Java code to format the data files, evaluate and predict. The file enzml_java_code.tar.gz contains the Java code used to format database data to ARFF and XML formats, to execute cross and train-test (jackknife) evaluations and to record evaluation results to database. More information is included in the readme.txt file and the Javadoc files. The code can be used with a MySQL database. To use a different database software, other JDBC drivers might be required. [file 1471-2105-13-61-S5.gz › java_code/enzml2011/doc/uk/ac/ed/inf/enzml/mulan/learn/TrainTestExperimenterSerialized.html]

TrainTestExperimenterSerialized


---


|  |  |  |
| --- | --- | --- |
| **Overview** \| **Package** \| **Class** \| **Use** \| **Tree** \| **Deprecated** \| **Index** \| **Help** | |  |
| **PREV CLASS**   NEXT CLASS | **FRAMES**    **NO FRAMES**     **All Classes** |  |
| SUMMARY: NESTED \| FIELD \| CONSTR \| METHOD | DETAIL: FIELD \| CONSTR \| METHOD |  |


---


## uk.ac.ed.inf.enzml.mulan.learn Class TrainTestExperimenterSerialized

```
java.lang.Object
  uk.ac.ed.inf.enzml.Initialised
      uk.ac.ed.inf.enzml.mulan.learn.MulanCrossExperimenter
          uk.ac.ed.inf.enzml.mulan.learn.TrainTestExperimenter
              uk.ac.ed.inf.enzml.mulan.learn.TrainTestExperimenterSerialized
```

**All Implemented Interfaces:**: Initialisable

---

``` public class TrainTestExperimenterSerialized extends TrainTestExperimenter ```

Executes a train/test evaluation using a previously trained model saved on
file.

**Version:**
:   25 Oct 2010

**Author:**
:   Luna De Ferrari luna.deferrari-at-ed.ac.uk

---

**Field Summary**

**Fields inherited from class uk.ac.ed.inf.enzml.Initialised**

`m_initialisationIsCorrect`


**Constructor Summary**

`TrainTestExperimenterSerialized(java.lang.String experimentPropsFile,
java.lang.String arffRecordDb,
int trainArffRecordId,
int testArffRecordId,
MulanLearner learner,
java.lang.String trainedModelFile)`


| **Method Summary** | |
| --- | --- |
| `TrainTestEvaluator` | `getEvaluator()` |
| `java.lang.String` | `getTrainedModelFile()` |
| `static void` | `main(java.lang.String[] args)` |
| `void` | `setTrainedModelFile(java.lang.String trainedModelFile)` |

**Methods inherited from class uk.ac.ed.inf.enzml.mulan.learn.TrainTestExperimenter**

`evaluateAndSave, getTestArffRecord`

**Methods inherited from class uk.ac.ed.inf.enzml.mulan.learn.MulanCrossExperimenter**

`crossEvaluateAndSaveResults, evaluateAndSave, getExperimentDbManager, getLearner, getResultsSaver, getTrainArffRecord`

**Methods inherited from class uk.ac.ed.inf.enzml.Initialised**

`initialisationIsCorrect, setInitialisationIsCorrect`

**Methods inherited from class java.lang.Object**

`equals, getClass, hashCode, notify, notifyAll, toString, wait, wait, wait`

**Constructor Detail**

### TrainTestExperimenterSerialized

```
public TrainTestExperimenterSerialized(java.lang.String experimentPropsFile,
                                       java.lang.String arffRecordDb,
                                       int trainArffRecordId,
                                       int testArffRecordId,
                                       MulanLearner learner,
                                       java.lang.String trainedModelFile)
```


**Method Detail**

### getEvaluator

```
public TrainTestEvaluator getEvaluator()
```

:   **Overrides:**: `getEvaluator` in class `TrainTestExperimenter`

---


### getTrainedModelFile

```
public java.lang.String getTrainedModelFile()
```

---


### setTrainedModelFile

```
public void setTrainedModelFile(java.lang.String trainedModelFile)
```

---


### main

```
public static void main(java.lang.String[] args)
```


---


|  |  |  |
| --- | --- | --- |
| **Overview** \| **Package** \| **Class** \| **Use** \| **Tree** \| **Deprecated** \| **Index** \| **Help** | |  |
| **PREV CLASS**   NEXT CLASS | **FRAMES**    **NO FRAMES**     **All Classes** |  |
| SUMMARY: NESTED \| FIELD \| CONSTR \| METHOD | DETAIL: FIELD \| CONSTR \| METHOD |  |


---
